# Supplementary material for: Hybrid Materials with Antimicrobial Properties Based on Hyperbranched Polyaminopropylalkoxysiloxanes Embedded with Ag Nanoparticles
Source: Pharmaceutics. 2023 Mar 2;15(3):809. doi: 10.3390/pharmaceutics15030809 (PMC10057488; doi:10.3390/pharmaceutics15030809)
Supplement: Supplementary file 1 [file pharmaceutics-15-00809-s001.zip › pharmaceutics-2260701-SI.pdf]

Hybrid materials with antibacterial properties based on hyperbranched polyaminopropylalkoxysiloxanes embedded with Ag nanoparticles

Alexander Vasil'kov, Dmitry Migulin, Alexander Naumkin, Ilya Volkov, Ivan Butenko, Alexandre Golub, Vera Sadykova and Aziz Muzafarov.

Supporting Information

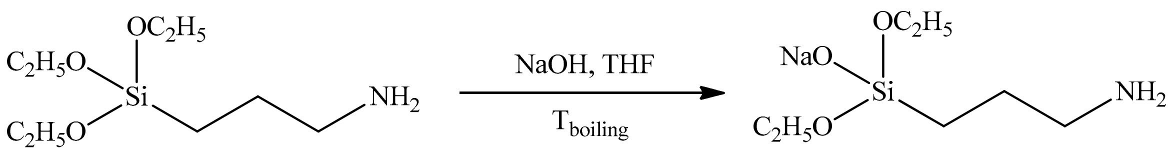

Figure S1. Scheme of synthesis of monosodium salts of (3-aminopropyl)diethoxysilanes.

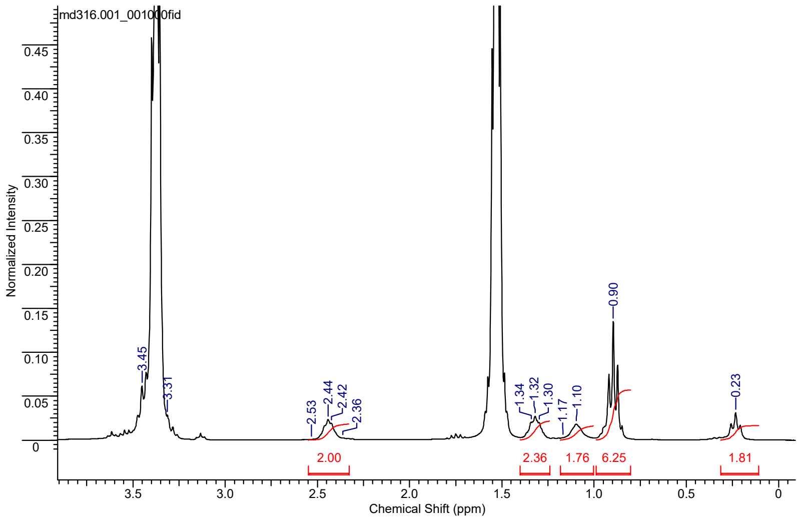

Figure S2. ^{1}H NMR Spectral data of Sodiumoxy-(3-Aminopropyl)diethoxysilane.

^{1}H NMR (300 MHz, THF) δ: 2.53-2.36 ppm (m, 2H ,-C$\underline{H}_2$-NH$_2$), 1.34-1,30 ppm (m, 2H, -C$\underline{H}_2$-CH$_2$-NH$_2$), 0.23 ppm (t, 2H, -Si-C$\underline{H}_2$-, J= 7.4 Hz), 0.9 ppm (t, 6H, C$\underline{H}_3$CH$_2$O-, J= 7 Hz), 3.45-3.31 ppm (m, 4H, CH$_3$C$\underline{H}_2$O-) 1.17-1.1 ppm (m, 2H, -N$\underline{H}_2$)

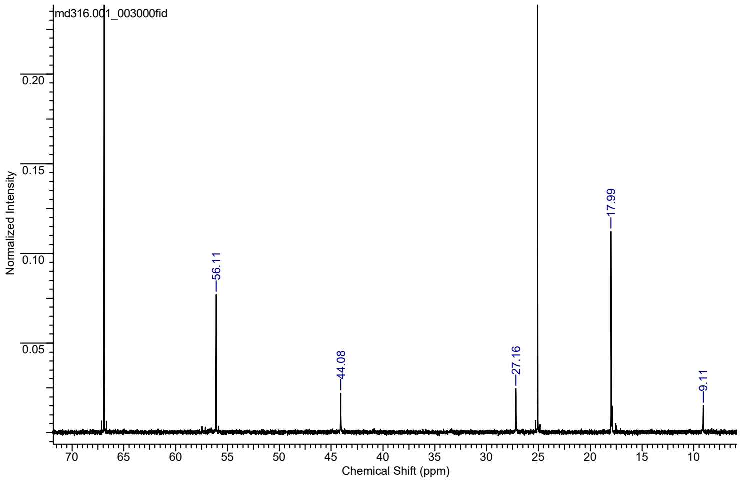

Figure S3. ^{13}C NMR Spectral data of Sodiumoxy-(3-Aminopropyl)diethoxysilane.

^{13}C NMR (THF) δ: 9.11 ppm (-Si-$\underline{C}H_2$-CH_2-), 27.16 ppm (-Si-CH_2-$\underline{C}H_2$-), 56.11 ppm (-CH_2-$\underline{C}H_2$-NH_2), 44.08 ($CH_3C\underline{H}_2O$-), 17.99 ppm ($C\underline{H}_3CH_2O$-).

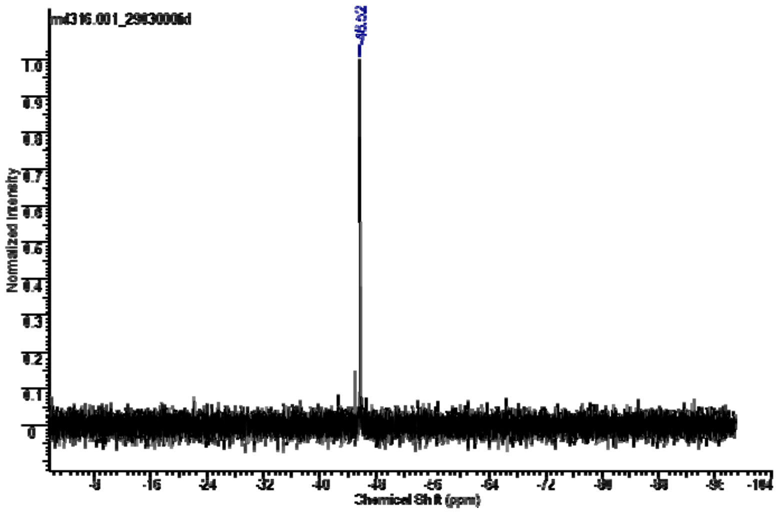

Figure S4. ^{29}Si NMR Spectral data of Sodiumoxy-(3-Aminopropyl)diethoxysilane.

^{29}Si (THF), δ: -46.52 ppm.

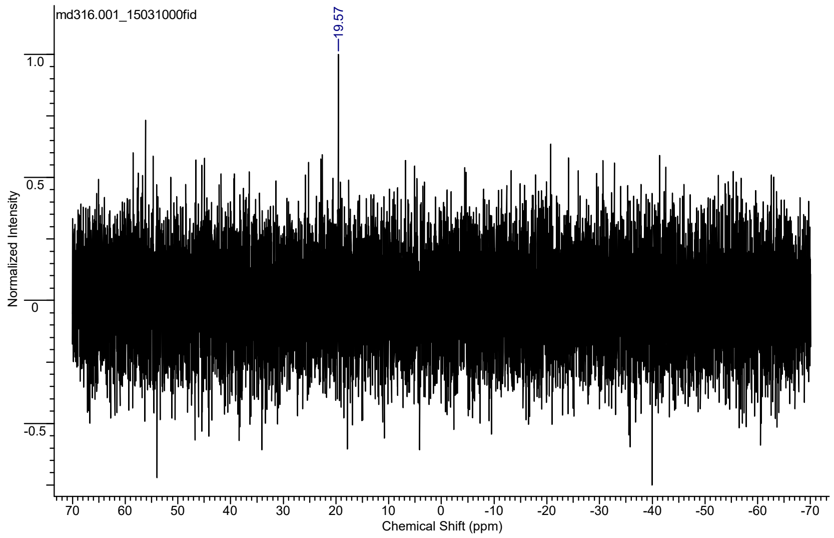

Figure S5. ^{15}N NMR Spectral data of Sodiumoxy-(3-Aminopropyl)diethoxysilan.

^{15}N (THF), δ: 19.6 ppm ($-NH_2$).

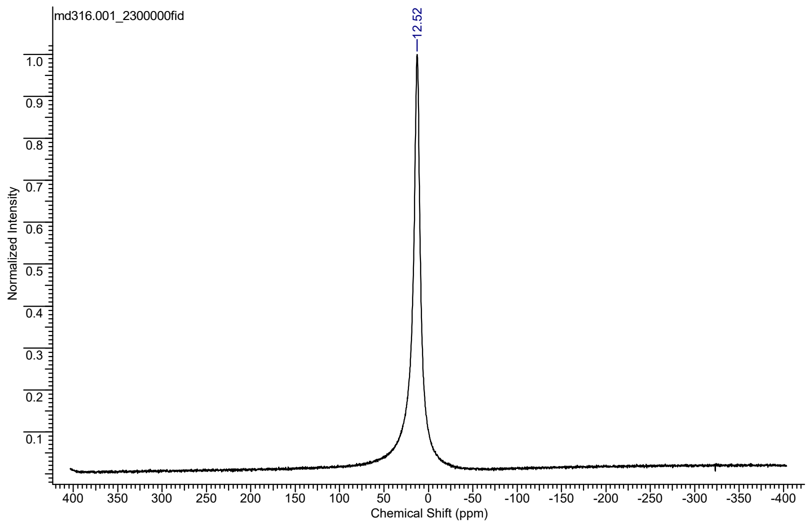

Figure S6. ^{24}Na NMR Spectral data of Sodiumoxy-(3-Aminopropyl)diethoxysilane.

^{23}Na (THF) 12.5 ppm.

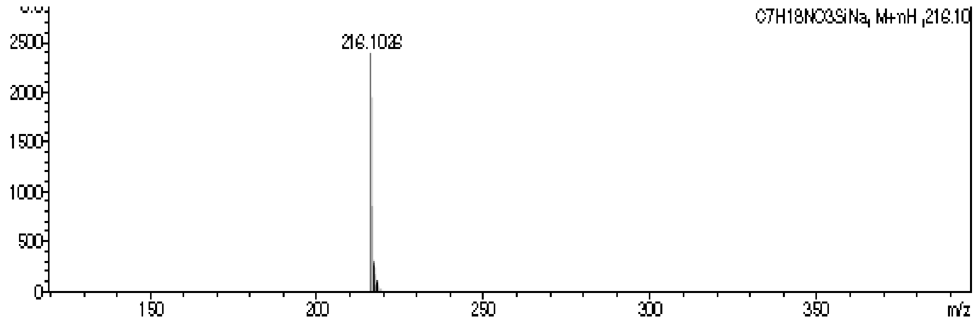

Figure S7. Mass spectra of Sodiumoxy-(3-Aminopropyl)diethoxysilane: HRMS calcd for $C_7H_{18}NNaO_3Si$: 216.1026; found: [M+nH] = 216.1024.

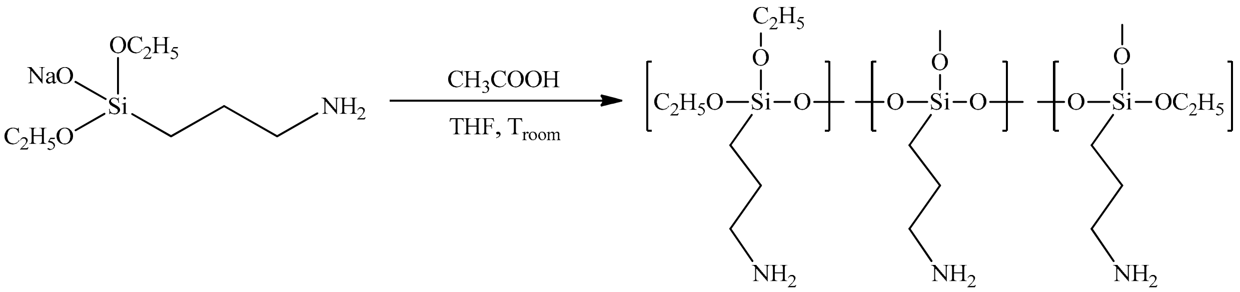

Figure S8. Scheme of synthesis of hyperbranched polyaminopropylethoxysiloxane_(HBPAPES)

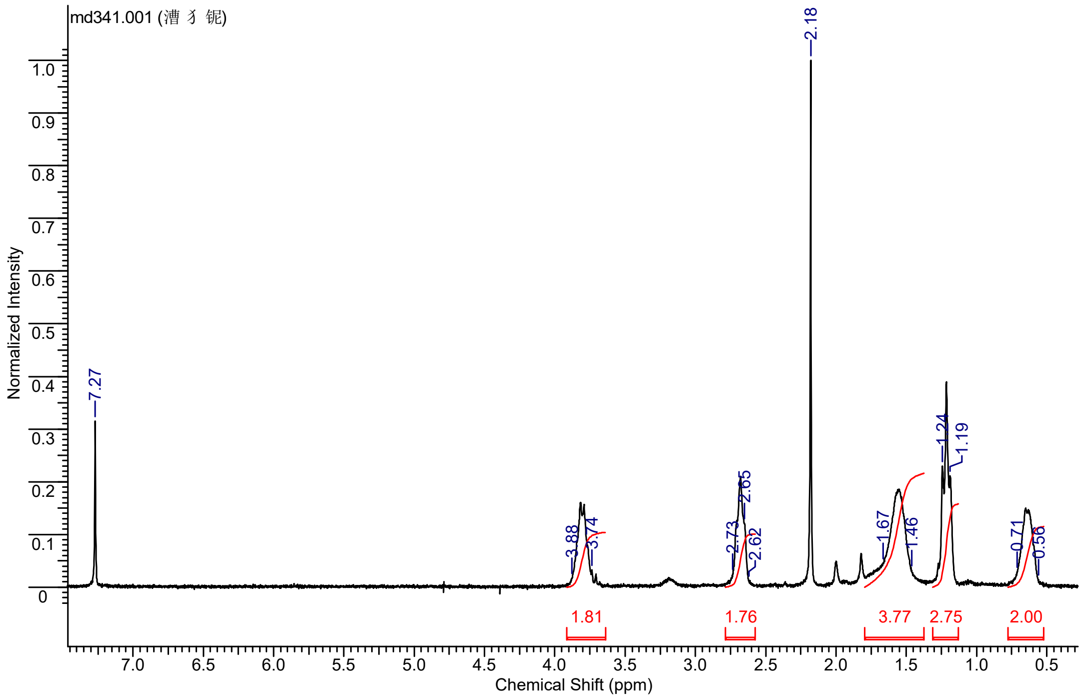

Figure S9. ^{1}H NMR Spectral data of polyaminopropylethoxysiloxane.

^{1}H NMR (300 MHz, $CDCl_3$) δ: 2.73-2.62 ppm (m, 2H ,-C<u>H</u>$_2$-NH_2), 1.67-1.46 ppm (m, 2H, -C<u>H</u>$_2$-CH_2-NH_2), 0.71-0.56 ppm (m, 2H, -Si-C<u>H</u>$_2$-), 1.24-1.19 ppm (m, 6H, C<u>H</u>$_3$$CH_2$O-), 3.88-3.74 ppm (m, 4H, CH_3C<u>H</u>$_2$O-), 1.67-1.46 ppm (m, 2H, -N<u>H</u>$_2$)

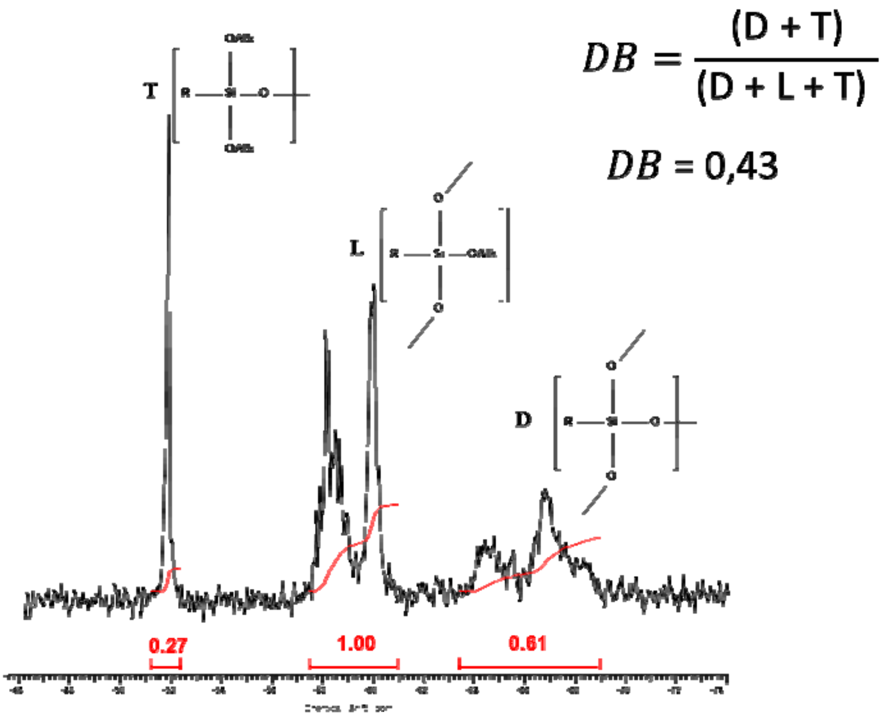

Figure S10. Degree of branching (DB) and ^{29}Si-NMR Spectral data of polyaminopropylethoxysiloxane with the addition of paramagnetic relaxation agent Chromium (III) Acetylacetonate.

^{29}Si (THF), δ: 51.89-52.3 ppm (m, R-Si(OCH$_3$)$_2$O-), 57.93-60.41 (m, R-Si(OCH$_3$)(O-)$_2$), 64.3-68.48 (m, R-Si(O-)$_3$).

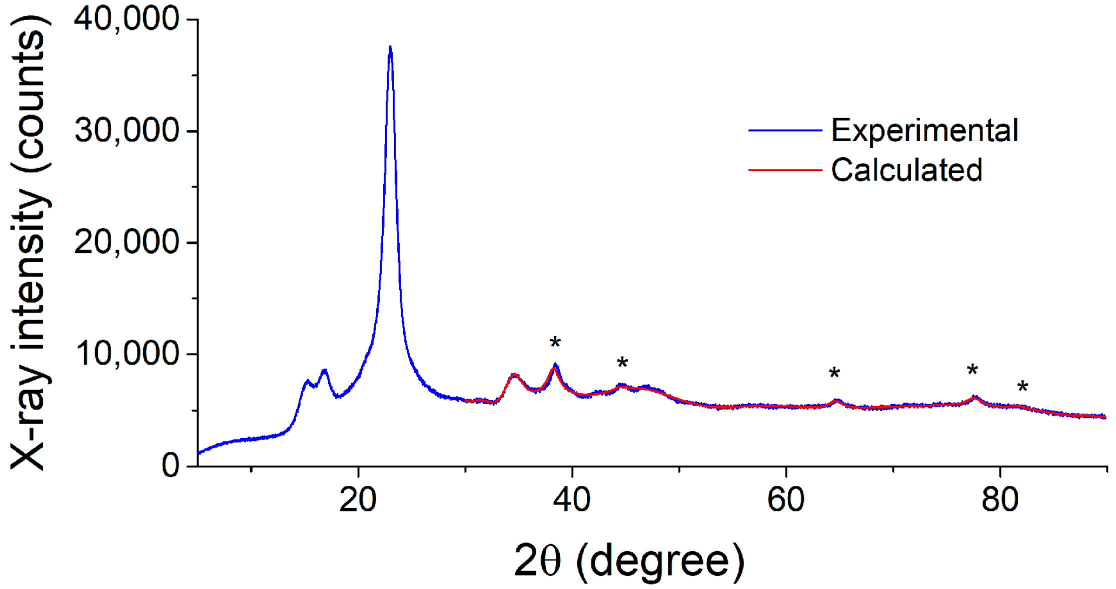

Figure S11. PXRD pattern of the Ag NPs/HBPAPES/cotton composite and its fit evidencing the presence of Ag nanoparticles. The reflections of metalic silver are marked by asterisks.

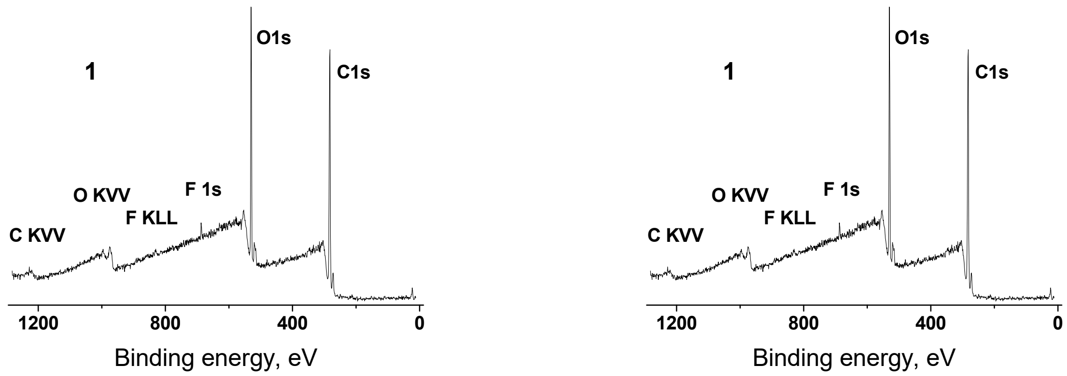

Figure S12. Survey XPS spectra of the cotton (**1**) and Ag NPs/HBPAPES/cotton (**2**) samples.

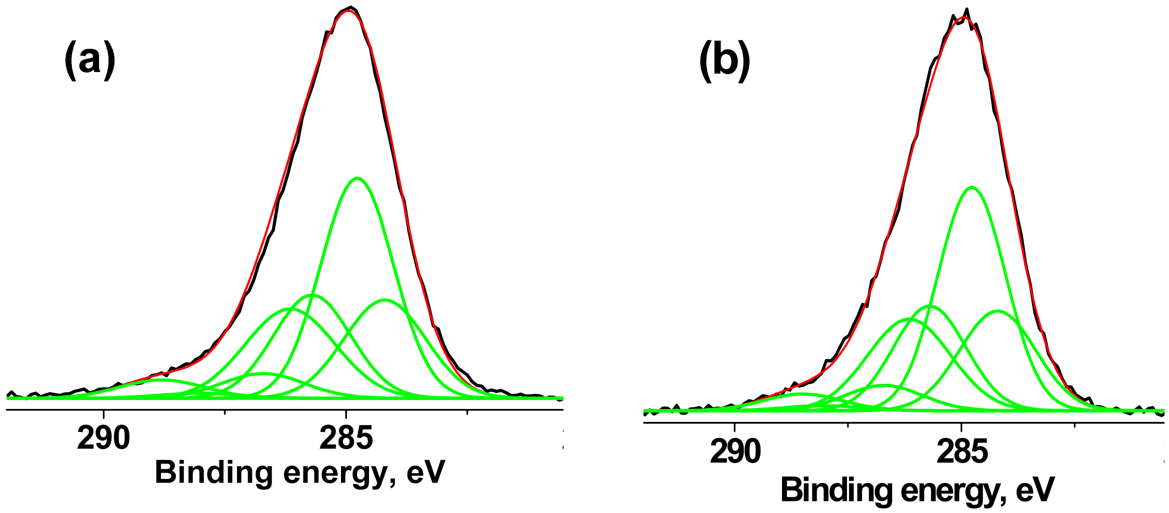

Figure S13. The C 1s spectra of the Ag NPs/HBPAPES/cotton sample: measured at U_b=0 (a) and 7 V (b) fitted in accordance to chemical structure formulas of silane and cotton.

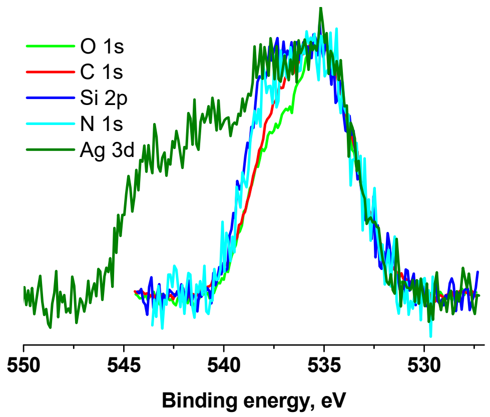

Figure S14. The O 1s, C 1s, Si 2p, N 1s and Ag 3d spectra of the Ag NPs/HBPAPES/cotton sample measured at U_b= -7 V. The figure demonstrates a coincidence of low-energy edges of all spectra which evidences on similar physical nature of low energy states.

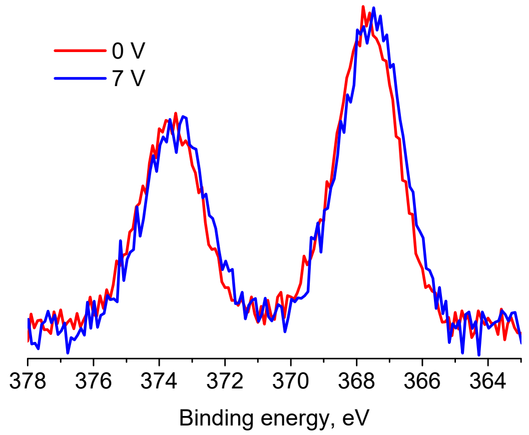

Figure S15. The Ag 3d spectra of the Ag NPs/HBPAPES/cotton sample measured at U_b = 0 V and U_b= 7 V.

Table S1. Quantification data (at. %) of the cotton and Ag NPs/HBPAPES/cotton samples recorded at various bias voltages

Sample	U_b	C	O	N	Ag	Si
Cotton	-7 V	66.1	33.9			
	0 V	67.3	32.7			
	7 V	67.8	32.2			
Ag NPs/HBPAPES/cotton	-7 V	74.1	18.6	2.7	0.6	4.1
	0 V	73.4	19.1	2.7	0.6	4.2
	7 V	74.9	17.9	2.4	0.5	4.3

Table S2. Parameters of components in C 1s and O 1s photoelectron spectra of the cotton sample: E_b – binding energy, W – peak width and I_{rel} – relative intensity.

		C 1s				O 1s			
	Peak	C1	C2	C3	C4	O1	O2	O3	O4
U_b	Parameter	C-C/C-H	C-OH	O-C-O	C(O)O	C(O*)O	C-OH	O-C-O	C(O)O*
7 V	E_b, eV	285.38	286.73	288.06	288.84	531.3	532.9	533.48	534.3
	W, eV	2.1	2.1	2.1	2.1	1.8	1.8	1.8	1.8
	I_{rel}	0.18	0.54	0.11	0.18	0.13	0.45	0.30	0.13
0 V	E_b, eV	285.38	286.73	288.06	288.76	531.3	532.91	533.49	534.3
	W, eV	2.1	2.1	2.1	2.1	1.8	1.8	1.8	1.8
	I_{rel}	0.22	0.51	0.10	0.17	0.11	0.47	0.31	0.11
-7 V	E_b, eV	285.53	286.73	288.06	288.76	531.44	532.93	533.51	534.00
	W, eV	2.1	2.1	2.1	2.1	1.8	1.75	1.79	1.8
	I_{rel}	0.19	0.53	0.11	0.17	0.13	0.44	0.30	0.13

Table S3. Parameters of peaks in C 1s photoelectron spectra of Ag NPs/HBPAPES/cotton: E_b – binding energy, W – peak width and I_{rel} – relative intensity.

		C 1s							Si $2p_{3/2}$			N 1s		Ag $3d_{5/2}$	Ag $3d_{3/2}$
U_b	Group	C-Si	C-C/C-H	C-N	C-O-Si	C-OH	O-C-O	C(O)O	Si^{4+}	Si^{3+}	Si^{4+}*	C-NH_2	*	Ag^{2+}	Ag^{2+}
0 V	E_b, eV	284.2	284.8	285.7	286.2	286.7	288.0	288.8	102.4	104.1		399.1	400.9	367.7	373.7
	W, eV	1.68	1.5	1.60	1.85	1.7	1.7	1.75	1.8	1.8		1.92	1.92	1.97	2.04
	I_{rel}	0.18	0.36	0.18	0.18	0.05	0.01	0.04	0.9	0.1		0.81	0.10	0.59	0.41
7 V	E_b, eV	284.2	284.8	285.7	286.2	286.7	288.0	288.5	102.3	103.9	99.8	399.0	401.2	367.6	373.5
	W, eV	1.68	1.50	1.60	1.83	1.7	1.7	1.75	1.8	1.8	1.8	1.88	1.89	1.96	2.00
	I_{rel}	0.18	0.36	0.18	0.18	0.05	0.01	0.03	0.77	0.12	0.11	0.88	0.12	0.6	0.4

* - differential charging
